# Surrogate Cerebrospinal Fluid Biomarkers for Assessing the Efficacy of Gene Therapy in Hurler Syndrome

**DOI:** 10.3389/fneur.2021.640547

**Published:** 2021-05-13

**Authors:** Reiner F. Haseloff, Stephanie Trudel, Ramona Birke, Michael Schümann, Eberhard Krause, Cathy Gomila, Jean-Michel Heard, Ingolf E. Blasig, Jérôme Ausseil

**Affiliations:** ^1^Leibniz-Forschungsinstitut für Molekulare Pharmakologie, Berlin, Germany; ^2^INSERM U1043, Centre de Physiopathologie de Toulouse-Purpan, Université Toulouse III Paul Sabatier, Toulouse, France; ^3^Service de Biochimie, Institut Fédératif de Biologie, Centre Hospitalier Universitaire de Toulouse, Toulouse, France; ^4^INSERM U1088, Centre Universitaire de Recherche en Santé, Université de Picardie Jules Verne, Amiens, France; ^5^Institut Pasteur, Paris, France

**Keywords:** surrogate marker, cerebrospinal fluid, mucopolysaccharidosis, gene therapy, mass spectrometry

## Abstract

Mucopolysaccharidosis type I (MPS I) is caused by a deficiency of the lysosomal hydroxylase alpha-l-iduronidase (IDUA). The resulting accumulation of dermatan and heparan sulfate induces intellectual disabilities and pre-mature death, and only a few treatment options are available. In a previous study, we demonstrated the feasibility, safety, and efficacy of gene therapy by injecting recombinant adeno-associated viral vector serotype (AAV)2/5-IDUA into the brain of a canine model of MPS I. We report on a quantitative proteomic analysis of control dogs and untreated dogs with MPS I cerebrospinal fluid (CSF) that had been collected throughout the study in the MPS I dogs. Mass spectrometry (MS) analysis identified numerous proteins present at altered levels in MPS I CSF samples. Quantitative immunoblotting, performed on CSF from healthy controls, untreated MPS I dogs, and MPS I dogs early treated and late treated by gene therapy, confirmed the MS data for a subset of proteins with higher abundance (neuronal pentraxin 1, chitinase 3-like 1, monocyte differentiation antigen CD14, and insulin-like growth factor-binding protein 2). Scoring of the results shows that the expression levels of these proteins are close to those of the control group for dogs that underwent gene therapy early in life but not for older treated animals. Our results disclose four novel predictive biomarker candidates that might be valuable in monitoring the course of the neurological disease in MPS patients at diagnosis, during clinical follow-up, and after treatment.

## Introduction

In the field of neurological and neurodegenerative diseases, there is an increasing need for reliable, objective cerebrospinal fluid (CSF) biomarkers of disease severity, disease progression/regression, and response to treatment. Although neurological diseases are most prevalent in the elderly, they also affect children with inherited metabolic diseases. In particular, lysosomal storage disorders (LSDs) result in intellectual disabilities and pre-mature death (1). The mucopolysaccharidoses (MPSs) are a family of hereditary LSDs caused by deficiencies of the lysosomal enzymes required for the degradation of glycosaminoglycans. Interruption of the heparan sulfate degradation pathway results in various levels of central nervous system involvement. Mucopolysaccharidosis type 1 (MPS I, also referred to as Hurler syndrome; OMIM #607014) is caused by deficiency of the lysosomal hydrolase alpha-l-iduronidase (IDUA, EC3.2.1.76), which leads to the accumulation of dermatan sulfate and heparan sulfate. The disorder is usually diagnosed before the age of 5 years. The most severely affected children show profound intellectual disabilities before the age of 10, and the progression of MPS I results in multiple disabilities. When performed in early childhood, bone marrow transplantation and hematopoietic stem cell transplantation are somewhat effective in patients with MPS I or II (2). The cloning of all genes implicated in the various types of MPS enabled the production of recombinant enzymes for administration as intravenous enzyme replacement therapy in MPS patients with peripheral lesions. We previously assessed the feasibility, safety, and efficacy of intracerebral gene therapy using recombinant adeno-associated viral vector serotype 2/5 (AAV)2/5-IDUA in canine models of MPS I and MPS IIIB (3). Our data demonstrated that (i) vector injections were well-tolerated, (ii) the vector genomes and the therapeutic enzyme were broadly distributed within the brain, (iii) brain lesions were less severe, and (iv) glycosaminoglycan levels in brain extracts were normalized. We also observed that late gene therapy was less efficacious than gene therapy immediately after weaning—confirming that the window for treating these diseases is narrow.

These promising results prompted us to test this treatment in patients with MPS IIIB in a phase I/II study (4). Indeed, several clinical trials of gene therapy for MPS are underway (www.clinicaltrials.gov). However, reliable brain-related biomarkers are still lacking for MPS I and other types of MPS but would be very helpful for monitoring the efficacy of these novel therapies.

In this context, the primary objective of the present study was to identify individual proteins or specific combinations thereof in the CSF, the presence or concentration of which would specifically reflect the MPS I disease stage. We addressed this goal by analyzing CSF samples that had been collected throughout the study in the MPS I dogs. Samples from healthy control dogs and untreated MPS I dogs underwent an unbiased proteomic analysis. Quantitative mass spectrometry (MS) revealed several potential biomarkers of disease progression. These candidates might be useful in monitoring brain lesions in patients with MPS I and other types of MPS at diagnosis, during clinical follow-up, and—as a guide to therapeutic effectiveness—after treatment.

## Methods

### CSF Samples

CSF samples used in this work had been collected throughout the study in the MPS I dogs, and all animal experiments have been described and published previously (3). Full details of how homozygous dogs carrying the recessive mutation underlying canine MPS I were bred (5) and diagnosed, AAV vector structure, their surgery, and vector administration and sacrifice can be found in the original publication (3). The Institutional Animal Care and Use Committee of the National Veterinary School of Nantes and the University of Nantes approved the experiments, which were performed by authorized investigators.

The investigated dog groups, the individual dog reference, the age at treatment, the immunosuppressive treatment (combination of mycophenolate mofetil (800 mg/m^2^/day) and a weekly-adjusted dose of cyclosporine to maintain cyclosporinemia above 300 ng/ml) and the pathology marker are summarized in Table 1. These data were reported by Ellinwood et al. (3).

**Table 1:** Overview of the study groups, treatment conditions, and disease marker.

**Disease and treatment**	**Dog ref**.	**Age at injection (months)**	**IS**	**Mean storage lesion score**
Untreated healthy control dogs	N1	–	–	1
	N2	–	–	1
	N3	–	+	1
MPS I dogs untreated	D11	–	+	3
	D18	–	–	3
	D22	–	–	3
MPS I dogs treated early	D30	4.1	+	1.2
	D31	4.3	+	1.5
	D37	5.2	+	1.2
MPS I dogs treated late	D15	8.9	+	2.7
	D17	10.0	+	3
	D25	8.3	+	3

*Information about the age (in months) at treatment (“age at injection”), immunosuppressive treatment (IS), and the mean cell vacuole (storage lesion) score described by Ellinwood et al. (3). MPS I, mucopolysaccharidosis type I*.

### Collection and Pre-treatment of CSF Samples

Samples of CSF (0.5–1 ml) from healthy control dogs and untreated, early-treated, and late-treated MPS I were collected, centrifuged within one hour, and stored at −80°C in polypropylene tubes (Sarstedt, Nümbrecht, Germany), in accordance with the international biobanking consensus guidelines optimized for CSF proteomics (6).

### Identification of Proteins Using Quantitative Mass Spectrometry

The proteomic analysis has been described previously (7, 8). Briefly, CSF samples were centrifuged (2,000 × g for 10 min), and proteins were precipitated with ice-cold acetone. The proteins were pelleted by centrifugation (10,000 × g for 30 min at 4°C), and the protein pellet was washed with ice-cold 90% acetone. The samples were briefly vortexed and then centrifuged again, and the supernatant was discarded. The protein pellets were dried for 30 min at room temperature, and the proteins were resuspended in water. Cysteine residues were reduced using dithiothreitol (10 mM, for 15 min, at 60°C) and alkylated (55 mM iodoacetamide, for 15 min, at 20°C) in the dark. Proteins were separated electrophoretically on precast Novex Tris/Glycine 4–20% gels (Life Technologies, Darmstadt/Germany). Proteins were visualized using Coomassie Brilliant Blue G-250.

Whole gel lanes were cut into slices of equal size (1 mm), and the excised gel slices were subjected to protein digestion for subsequent MS. During in-gel tryptic digestion in the presence of either ${\text{H}}_{2}^{16}$O or ${\text{H}}_{2}^{18}$O (Sigma, Taufkirchen/Germany), peptides were labeled with the stable isotope. After peptide extraction, samples from paired gel slices were combined immediately prior to analysis by nanoLC-MS/MS.

The nanoLC-MS/MS analyses were performed on a LTQ-Orbitrap XL mass spectrometer (Thermo Scientific, Dreieich/Germany) equipped with a reverse-phase capillary liquid chromatography system (Eksigent 2D nanoflow LC system, Axel Semrau GmbH, Sprockhövel/Germany). The MASCOT server (version 2.2., Matrix Sciences Ltd., London, UK) and the Proteome Discoverer 2.4 (Thermo Scientific) were used to search the Swiss-Prot (mammals)/TrEMBL (dog) database (release 2020_02). A maximum of two missed cleavages was allowed, and mass tolerance of precursor and fragment ions was set to 10 ppm and 0.5 Da, respectively. Acrylamide or carbamidomethyl modification of cysteine, methionine oxidation, acetylation (N-terminal), and C-terminal ^18^O_1_ and ^18^O_2_ isotope labeling were considered possible modifications. Based on decoy database searches, the false-positive rate was estimated to be below 1%. Protein quantifications were accomplished using the Mascot Distiller Quantitation Toolbox (version 2.2.1.2, Matrix Science Ltd.), based on calculations of isotope intensity ratios of at least two tryptic peptides with individual MASCOT scores indicating homology. Relative protein ratios were calculated from the intensity-weighted average of all peptide ratios.

### The Alpha-l-Iduronidase Assay

The level of IDUA activity was assayed as described previously (9); the method was adapted for use in concentrated CSF. Proteins in 300 μl of CSF were titrated and concentrated six times on Spin-X. IDUA catalytic activity was measured in 50 μl of concentrated CSF by adding 25 μl of 4 mM fluorescent 4-methylumbelliferyl-alpha-iduronide for 18 h at 37°C followed by optic deviation lecture (excitation 355 nm, emission 440 nm). Each CSF sample was tested twice in two runs of evaluation. Results are expressed in nanomoles per hour per milliliter of CSF. The limit of quantification was <5 nmol/h/ml of CSF.

### Quantitative Immunoblotting

As suggested for CSF (10), total protein (determined using the Pierce 660 nm Protein Assay, Thermo Scientific) served as a loading control for immunoblotting. Proteins were separated electrophoretically and blotted onto a nitrocellulose membrane (Hybond™-ECL; GE Healthcare, Freiburg/Germany). The membrane was blocked overnight at 4°C with 5% (w/v) powdered milk in 25 mM Tris pH 7.4, 300 mM NaCl, 0.01% (v/v) Tween 20 (TBST) and incubated first with the respective primary antibody in TBST for 90 min and then with corresponding goat antirabbit/antimouse IgG (H + L) horseradish peroxidase-conjugated secondary antibodies (Thermo Scientific) for 45 min at room temperature. Horseradish peroxidase activity was quantified in a LumiAnalyzer with chemiluminescence detection reagents (GE Healthcare). The primary antibodies were rabbit antihuman neuronal pentraxin 1 (NP1, Sigma, Taufkirchen/Germany), rabbit antihuman insulin-like growth factor-binding protein 2 (IGFBP2, Thermo Scientific), rabbit antihuman chitinase-3-like 1 (Ch3L1, Biorbyt, Cambridge/UK), and mouse antihuman monocyte differentiation antigen CD14 (clone TÜK4, BioRad, Feldkirchen/Germany). Immunoblots were scanned and quantified densitometrically (unblinded) using Image J software v1.47 (https://imagej.nih.gov/ij/index.html, National Institutes of Health, Bethesda, MD/USA).

## Results

### Quantification of IDUA Activity in the CSF of MPS I Dogs

We first assayed IDUA activity in the CSF samples, in order to determine whether the disease correction observed in the brain of treated MPS I dogs was reflected by changes in the cerebrospinal compartment. The activity was measured in three individual animals (*n* = 3) from each of the four groups: healthy controls, untreated MPS I dogs, and early-treated and late-treated MPS I dogs. As expected, lack of IDUA activity was detected in CSF from MPS I dogs, whereas CSF from healthy controls exhibited high levels of IDUA activity (Table 2). The IDUA assay revealed that, although IDUA activity in CSF from treated MPS I dogs did not reach the level observed in normal dogs, it was significantly higher than in untreated MPS I dogs. Levels of IDUA activity tended to be higher in early-treated MPS I dogs than in late-treated MPS I dogs; however, the difference was not statistically significant.

**Table 2:** IDUA activity and overall score in CSF.

**Disease and treatment**	**Dog ref**.	**CSF IDUA activity (nmol/h/ml)**	**Overall score**
Untreated healthy control dogs	N1	33.2 (0.3)	1
	N2	36.1 (0.1)	1
	N3	35.9 (0.2)	1
MPS I dogs untreated	D11	< LOQ	2
	D18	< LOQ	2.8
	D22	< LOQ	2.3
MPS I dogs treated early	D30	7.0 (0)	1.4
	D31	5.4 (0.1)	1.3
	D37	6.7 (0.2)	1.4
MPS I dogs treated late	D15	5.0 (0.1)	2.7
	D17	3.9 (0.1)	2.7
	D25	4.9 (0)	2.5

*The overall score obtained from our quantitative immunoblot and the IDUA activity detected in concentrated CSF (in nmol/h/ml of CSF). Values are means (SEM) of at least two independent technical replicates. LOQ in CSF = 5 nmol/h/ml. CSF, cerebrospinal fluid; IDUA, alpha-l-iduronidase; MPS I, mucopolysaccharidosis type I; LOQ, limit of quantification*.

### Identification and Verification of CSF Biomarkers

Proteins of low abundance are more likely to be suitable as specific and reliable biomarkers, thus of much higher relevance for rare diseases, such as MPSs, Consequently, pooling of the samples for proteomic profiling was enforced by the relatively low protein amounts in dog CSF samples. Depletion of highly abundant proteins was avoided since depletion protocols are often accompanied by an inadvertent loss of material (11). However, separation of the sample by SDS gel electrophoresis allows to excise the albumin band which has been shown to account for up to 60% of total CSF protein (12) (Supplementary Figure 1). This separation also provides, in addition to protein identities, information on the molecular weight and may help to distinguish functional proteins from complexes or fragments. Approximately 500 proteins were identified in CSF. After applying a threshold of 2.0 to the heavy-to-light (H/L) *I*_O18pep_/*I*_O16pep_ ratio (11), 18 proteins were considered to be present at higher levels in the MPS I CSF sample than in healthy controls. The fold difference ranged from 2.4 to >50 (Table 3). Fifty-seven proteins were found at lower abundance in MPS 1 animals; a few examples are given in Table 3.

**Table 3:** Proteins changed in abundance in cerebrospinal fluid of MPS 1 dogs compared with healthy control dogs.

**Accession**	**Protein**	**M.W**.	**Score**	**H/L**
E2R478	Neuronal pentraxin 1	47177	328	>50
P25311	Zinc-alpha-2-glycoprotein	34259	321	27.1
Q3MHN5	Vitamin D-binding protein	53342	93	13.0
F1P8G0	Fibrinogen gamma chain	49317	139	12.2
D3YJ60	Chitinase 3-like 1	42623	43	11.5
F1PGM1	Complement C3	176139	493	10.6
J9NT12	Fibrinogen like 1	36308	113	9.3
G1K2B9	von Willebrand factor	308522	69	8.4
P18649	Apolipoprotein E	37224	962	7.6
P19006	Haptoglobin	36457	149	7.5
P01785	Ig heavy chain V region MOO	12703	90	7.2
A0A1S7J0A8	Monocyte differentiation antigen CD14	39897	50	6.8
T2KEN6	Pentaxin (C-reactive protein)	25383	91	6.3
E2R833	Leucine-rich alpha-2-glycoprotein 1	38987	172	5.1
F1PAF3	Hepatocyte growth factor activator	77450	116	4.7
P18065	Insulin-like growth factor-binding protein 2	34814	144	3.9
A0A5F4CMK5	Neural cell adhesion molecule 2	93031	99	3.0
P33703	Beta-2-glycoprotein 1	38403	390	2.4
A0A5F4BV75	Contactin 1	112977	921	0.2
F1PCR2	Mannose receptor C type 2	170178	302	0.1
J9NZW8	Seizure-related 6 homolog-like 2	97907	174	0.1
P36955	Pigment epithelium-derived factor	46312	822	0.03
J9NZL6	Reelin	387929	289	0.02

*Underlined proteins were selected for further experiments. Accession, accession number in UniprotKB; M.W., molecular weight; H/L, ratio of MS signal intensities of peptides originating from MPS I vs. healthy controls (=fold change in protein abundance); Score, Mascot (search engine) score*.

We next used quantitative immunoblotting to verify the MS data for a subset of candidate biomarkers, with a focus on proteins known to have a role in neuron biology, neuroinflammation, neurodegenerative diseases, and/or pathophysiology in general: NP1, Ch3L1, CD14, and IGFBP2. Pooled protein samples from the three healthy controls, the three untreated MPS I dogs, and the six treated (early and late) MPS I dogs were analyzed to confirm the higher abundance of these proteins and to check whether gene therapy normalized the respective protein levels in the CSF of MPS I-treated dogs. Special consideration was given to the exact determination of each sample's protein concentration, a pre-condition for the use of total protein as a protein standard for CSF (10) (Supplementary Figure 1). All selected proteins were more abundant in MPS I samples than in control samples (Supplementary Figure 2). Interestingly, an additional isoform of NP1 was detected in MPS I dogs. The quantitative analyses also demonstrated that levels of all four proteins were lower in treated MPS I dogs than in untreated MPS I dogs; approximate control values were achieved for IGFBP2 (Supplementary Figure 2).

### Evaluation of Each Biomarker's Ability to Predict Disease Correction

**Figure 1:**
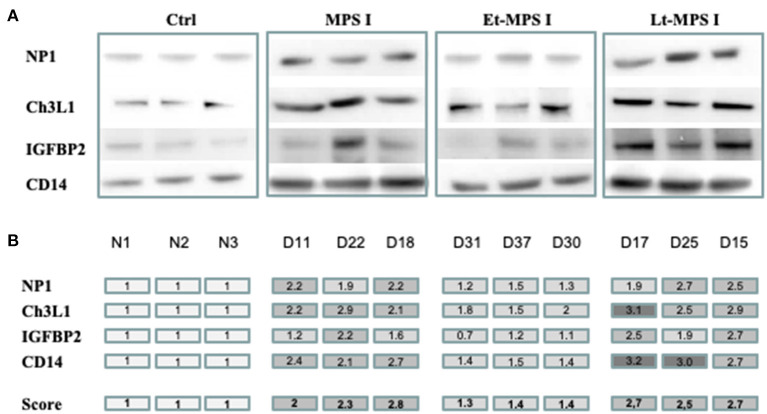
Evaluation of each biomarker's predictive value for disease correction by quantitative immunoblotting. Equal concentrations of individual CSF samples (*n* = 3) of healthy control dogs, untreated MPS I dogs, and early- and late-treated MPS I dogs were analyzed by Western blotting. Using ImageJ software, each band was scored semiquantitatively from 1 to 3 (1 = the value of the control). **(A)** Western blot images from each CSF sample for NP1, Ch3L1, CD14, and IGFBP2 proteins. **(B)** A color code indicates scores for each protein. An overall score (the average of the four protein scores) is shown in the bottom row. NP1, neuronal pentraxin 1; Ch3L1, chitinase 3-like 1; IGFBP2, insulin-like growth factor-binding protein 2; CD14, monocyte differentiation antigen CD14; Ctrl, healthy control; Et-MPS I, early-treated MPS I dogs; Lt-MPS I, late-treated MPS I dogs.

Using the immunoblot of each individual dog's CSF sample, the relative concentrations of NP1, Ch3L1, CD14, and IGFBP2 proteins were quantified (Figure 1A), and an overall score (the average of the four individual protein scores) was calculated (Figure 1B). Early-treated MPS I dogs had scores that were closest to those in healthy controls, whereas similar, high scores were observed in MPS I dogs and late-treated MPS I dogs (Table 1 and Figure 1). Within the early-treated group, the scores varied markedly from one dog to another; relative to control values, the score ranged from 0.7 to 2.0. Conversely, the individual scores for late-treated MPS I dogs ranged from 1.9 to 3.2 relative to controls.

## Discussion

Biomarker research is an expanding, emerging field which constitutes an important aspect in the development of 3P (predictive, preventive, personalized) medicine (13). However, reliable brain-related biomarkers for MPS I and other types of MPSs are lacking—as can be seen for many rare diseases. Biomarkers of MPS would facilitate the development and validation of novel therapies. Currently, eight gene therapy clinical trials in MPS are recruiting, one in MPS I (NCT03580083), two in MPS II (NCT03566043, NCT04571970), four in MPS III (NCT04088734, NCT02716246, NCT03315182, NCT04201405) and one in MPS VI (NCT03173521) (www.clinicaltrials.gov).

Cerebrospinal fluid is close to the disease site in neurodegenerative disorders, providing several advantages as a source of biomarkers compared with more distal fluids such as plasma (14, 15). The current study describes first steps in a workflow for identifying CSF biomarkers of MPS I based on the analysis of CSF samples from healthy control dogs, untreated MPS I dogs, and MPS I dogs that had undergone gene therapy. As our previous data had shown that pathology markers were not normalized or only partially recovered in MPS I dogs that received the treatment after the age of 7 months (3), we deliberately studied early- and late-treated MPS I dogs.

Detection of IDUA activity in the CSF confirmed the efficacy of vector delivery and gene transfer to the brain. The IDUA activity in the CSF of MPS I-treated dogs is about 20–30% of that in healthy controls; our previous study shows that the IDUA activity in the brain of treated dogs was equivalent to or higher than that found in controls. This result indicates that only a fraction of the delivered enzyme enters the CSF and is consistent with our published data on intracerebral gene therapy in MPS IIIB children; the α-*N*-acetylglucosaminidase enzymatic activity in CSF was 15–20% of that in unaffected children (4).

The quantitative proteomic approach yielded a variety of proteins with a greater abundance in MPS I dogs (Table 3), but none of them exhibited a clear relationship to MPS I. Our selection of proteins for verification was based on (i) our proteomic results, (ii) the proteins' known associations with neuron biology, neuroinflammation, neurodegenerative diseases and/or pathophysiology, and (iii) the availability of reliable antibodies for the respective proteins.

The top-ranking protein with a fold change >50 is NP1, encoded by the *NPTX1* gene. This member of the pentraxin family is preferentially expressed in the brain and is involved in neurodegeneration (16) and glutamate receptor internalization (17). It is found in the pre- and postsynaptic compartments of excitatory synapses (17). Neuronal pentraxin 1 has been recently described as a synapse-derived plasma and CSF biomarker for Alzheimer's disease (AD). A decrease in CSF levels of NP1 has been found in AD (18) while plasma NP1 levels were abnormally high in older adults with mild cognitive impairment and even higher in the subset of individuals who progressed to early-stage AD (19). Elevated expression of an alternative isoform of NP1 was recently observed in a mouse model of Sandhoff disease (another LSD) (20). The additional isoform of NP1 in both untreated and treated MPS I dogs (Supplementary Figure 2), could be an explanation for the high value of the fold change found in MS. Unfortunately, varying isoforms could not be detected by MS, which is explained by the reported data that point to a truncated form of NP1. Nevertheless, the demonstration of Hooper et al. that *Np1* knockout was associated with more normal behavioral patterns and longer survival in the mouse model of Sandhoff disease suggests that NP1 or its alternative isoforms contribute to disease progression.

Our proteomic approach identified several inflammation-linked proteins with a higher abundance in CSF from MPS I dogs. Notably, this refers to CD14, Ch3L1, and IGFBP2. For these proteins, we confirmed the normalization of their levels in treated MPS I dogs (Figure 1 and Supplementary Figure 2). CD14 (particularly soluble fragments, sCD14) has been mentioned as a possible marker of bacterial infections, including sepsis (21); however, it probably needs a combination with other markers to provide a reliable diagnosis (22). Elevated levels of Ch3L1 and sCD14 have been identified as promising immunomarkers in CSF samples from children with inflammatory disorders (23). The assembly of proteins relevant in inflammation also includes C-reactive protein, zinc-alpha-2-glycoprotein, neural cell adhesion molecule 2 (24), vitamin D-binding protein (25), fibrinogen-like protein 1 (26, 27), and hepatocyte growth factor activator (28).

Both sCD14 and Ch3L1 are considered to be microglial markers, which coincides with our data that microglial activation has a central role in the physiopathology of MPS (29, 30). Ch3L1 (in humans also named YKL-40) can be assumed as an almost universal marker of diseases: for neural pathologies, it has been suggested as a biomarker of multiple sclerosis (31), traumatic brain injury (32), Alzheimer's disease (33), amyotrophic lateral sclerosis (34), iatrogenic and sporadic Creutzfeldt-Jakob disease (35), or bipolar disorder (36)—not to mention various pathologies in peripheral organs.

IGFBP2 has been suggested as a potential biomarker for glioblastoma, prostate cancer (37), colorectal cancer (38), and lupus nephritis (39). In HIV-positive individuals, IGFBP2 levels are positively correlated with the plasma level of IL-6 and the CSF level of tumor necrosis factor alpha (40).

The overall scores calculated for NP1, Ch3L1, CD14, and IGFBP2 almost reached control values in early-treated MPS I dogs, while the corresponding values for late-treated MPS I dogs were similar to those for untreated MPS I dogs—suggesting that the overall score can predict the efficacy of gene therapy in the canine model of Hurler syndrome. However, our results also show that the quantitative score for an individual protein is not predictive of disease correction. For example, the individual Ch3L1 score is 2.0 (a value typically observed in untreated MPS I dogs) at D30 (in the early-treated dogs), while the overall score for the four proteins at D30 is 1.4—much closer to control values. These observations confirm the higher adequacy of a set of biomarkers for assessing the course of the disease.

## Conclusions

Considering the lack of reliable brain-related biomarkers of MPS and ongoing clinical trials, this study demonstrates that the determination of the CSF levels of NP1, Ch3L1, CD14, IGFBP2, possibly also of other upregulated proteins identified here could be helpful for assessing the course of the disease in infants having received gene therapy. Further validation of these biomarker candidates, e.g., by investigating the correlation between CSF and plasma/serum levels in MPS patients, may offer a less-invasive means of routine monitoring.

## Data Availability Statement

Datasets are available on request: data supporting the conclusions of this article will be made available by the authors (RH), without undue reservation.

## Ethics Statement

The animal study was reviewed and approved by The Institutional Animal Care and Use Committee of the National Veterinary School of Nantes and the University of Nantes approved experiments, which were performed by authorized investigators.

## Author Contributions

RB and CG performed the biochemical experiments. EK and MS performed the mass spectrometry experiments. MS and RH analyzed MS data. J-MH and IB conceived and designed the study. RH, ST, and JA conceived and designed the experiments, interpreted the data, and wrote the manuscript. All authors read and approved the final manuscript.

## Conflict of Interest

The authors declare that the research was conducted in the absence of any commercial or financial relationships that could be construed as a potential conflict of interest.

## Abbreviations

AAV: adeno-associated virus
AD: Alzheimer's disease
CD14: monocyte differentiation antigen CD14
Ch3L1: chitinase-3-like 1
CSF: cerebrospinal fluid
IDUA: alpha-l-iduronidase
IGFBP2: insulin-like growth factor-binding protein 2
LSD: lysosomal storage disorder
MPS: mucopolysaccharidose
MS: mass spectrometry
NP1: neuronal pentraxin 1
TLR: toll-like receptor.
