# Supplementary figures and images for: Surrogate Cerebrospinal Fluid Biomarkers for Assessing the Efficacy of Gene Therapy in Hurler Syndrome
Source: Front Neurol. 2021 May 13;12:640547. doi: 10.3389/fneur.2021.640547 (PMC8155356; doi:10.3389/fneur.2021.640547)

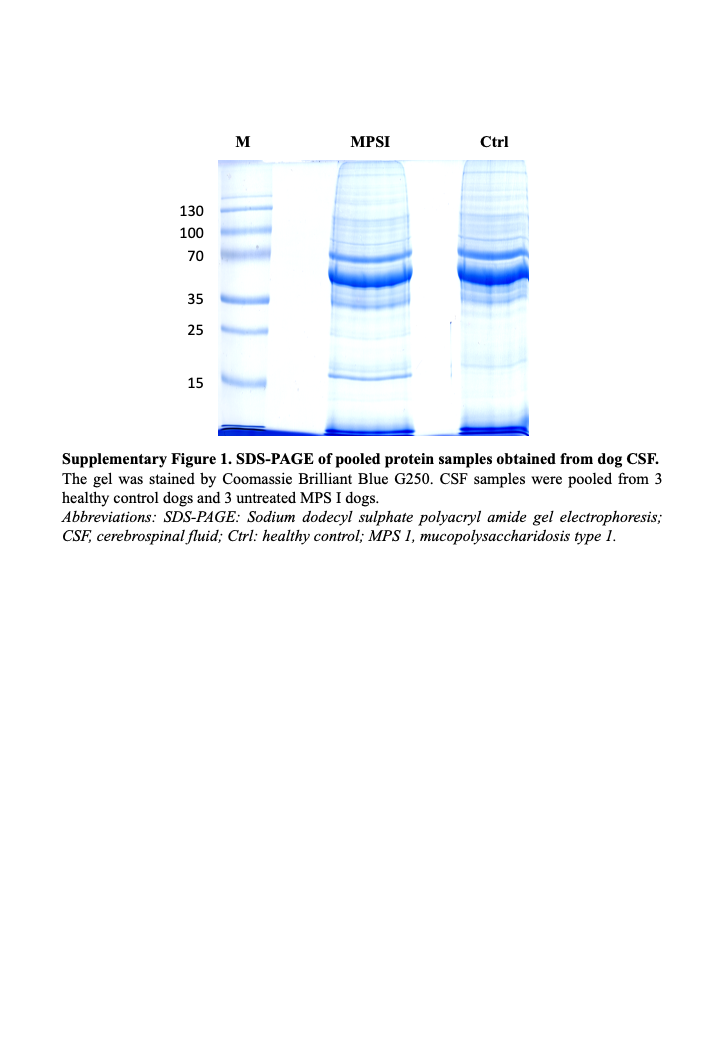

Supplement: Supplementary file 1 [file Image_1.tiff]

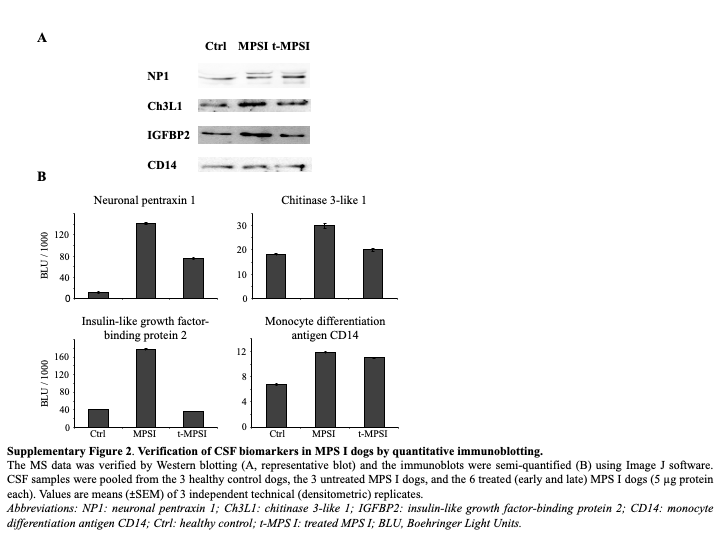

Supplement: Supplementary file 2 [file Image_2.tiff]
